# Anti-inflammatory effect of *Barringtonia angusta* methanol extract is mediated by targeting of Src in the NF-κB signalling pathway

**DOI:** 10.1080/13880209.2021.1938613

**Published:** 2021-06-30

**Authors:** Minkyeong Jo, Jongsung Lee, Han Gyung Kim, Jin Kyeong Kim, Haeyeop Kim, Kon Kuk Shin, Tran The Bach, Sang Mi Eum, Jong Sub Lee, Eui Su Choung, Yoonyong Yang, Kyung-Hee Kim, Gi-Ho Sung, Byong Chul Yoo, Jae Youl Cho

**Affiliations:** a Department of Integrative Biotechnology, Sungkyunkwan University, Suwon, Republic of Korea; b Research Institute of Biomolecule Control and Biomedical Institute for Convergence at SKKU (BICS), Sungkyunkwan University, Suwon, Republic of Korea; c Institute of Ecology and Biological Resources, Vietnam Academy of Science and Technology (VAST), Ha Noi, Vietnam; d International Biological Material Research Center, Korea Research Institute of Bioscience & Biotechnology, Daejeon, Republic of Korea; e DanjoungBio Co., Ltd, Wonju, Republic of Korea; f Biological and Genetic Resources Assessment Division, National Institute of Biological Resources, Incheon, Republic of Korea; g Proteomic Analysis Team, Research Institute, National Cancer Center, Goyang, Republic of Korea; h Department of Microbiology, Biomedical Institute of Mycological Resource, International St. Mary's Hospital and College of Medicine, Catholic Kwandong University, Incheon, Republic of Korea; i Division of Translational Science, Research Institute, National Cancer Center, Goyang, Republic of Korea

**Keywords:** Inflammation, gastritis, macrophages, inflammatory genes, signalling cascade

## Abstract

**Context:**

Among the plants in the genus *Barringtonia* (Lecythidaceae) used as traditional medicines to treat arthralgia, chest pain, and haemorrhoids in Indonesia, *Barringtonia racemosa* L. and *Barringtonia acutangula* (L.) Gaertn. have demonstrated anti-inflammatory activity in systemic inflammatory models.

**Objective:**

The anti-inflammatory activity of *Barringtonia angusta* Kurz has not been investigated. We prepared a methanol extract of the leaves and stems of *B. angusta* (Ba-ME) and systemically evaluated its anti-inflammatory effects *in vitro* and *in vivo*.

**Materials and methods:**

RAW264.7 cells stimulated with LPS or Pam3CSK4 for 24 h were treated with Ba-ME (12.5, 25, 50, 100, and 150 µg/mL), and NO production and mRNA levels of inflammatory genes were evaluated. Luciferase reporter gene assay, western blot analysis, overexpression experiments, and cellular thermal shift assay were conducted to explore the mechanism of Ba-ME. In addition, the anti-gastritis activity of Ba-ME (50 and 100 mg/kg, administered twice per day for two days) was evaluated using an HCl/EtOH-induced gastritis mouse model.

**Results:**

Ba-ME dose-dependently suppressed NO production [IC_50_ = 123.33 µg/mL (LPS) and 46.89 µg/mL (Pam3CSK4)] without affecting cell viability. Transcriptional expression of *iNOS*, *IL-1β*, *COX-2*, *IL-6*, and *TNF-α* and phosphorylation of Src, IκBα, p50/105, and p65 were inhibited by Ba-ME. The extract specifically targeted the Src protein by binding to its SH2 domain. Moreover, Ba-ME significantly ameliorated inflammatory lesions in the HCl/EtOH-induced gastritis model.

**Discussion and Conclusions:**

The anti-inflammatory activity of Ba-ME is mediated by targeting of the Src/NF-κB signalling pathway, and *B. angusta* has potential as an anti-inflammatory drug.

## Introduction

Inflammation is part of the immune system that maintains homoeostasis in the body in response to infection or injury (Ferrero-Miliani et al. 2007). Various kinds of immune cells, including macrophages, neutrophils, and dendritic cells, protect the body against invading pathogens. The process of inflammation is characterized by several symptoms, such as fever, pain, redness, and swelling (Fang et al. 2018; Chen, Deng et al. 2018).

The inflammatory response of immune cells typically progresses through inducers, sensors, mediators, and target tissues. When pathogens (viruses, bacteria, or fungi) invade a body or tissues are damaged, pattern recognition receptors on immune cells, such as toll-like receptors (TLRs), act as sensors that detect pathogen-associated molecular patterns and damage-associated molecular patterns (Stephens et al. 2019). Inflammatory cytokines such as interleukin (IL)-1, IL-6, and tumour necrosis factor (TNF)-α are then secreted to various target tissues and produce inflammatory responses (Medzhitov 2010; Kim, Kang et al. 2018). For example, when macrophages are activated by lipopolysaccharide (LPS) and TLR4, TLR4 activates the adaptor molecules myeloid differentiation primary response 88 (MyD88) and TIR-domain-containing adaptor-inducing interferon-β (TRIF) to trigger inflammatory signalling pathways, such as nuclear factor-κB (NF-κB) and activator protein 1 pathways (Kagan and Medzhitov 2006; Chen, Shao et al. 2018). When NF-κB signalling is turned on, the protein tyrosine kinase Src and IκB kinases are activated and induce the phosphorylation of IκB, which is bound to inactive NF-κB in unstimulated cells (Funakoshi-Tago et al. 2005). Phosphorylation of IκB leads to its degradation, and the released NF-κB translocates to the nucleus. NF-κB binding to specific motifs in the promoter regions of target genes results in transcription of inflammatory genes and cytokines encoding proteins such as inducible nitric oxide synthase (iNOS), cyclooxygenase (COX)-2, IL-1β, and TNF-α. In response to production of these inflammatory genes and cytokines, additional inflammatory mediators and cytokines such as nitric oxide (NO) and prostaglandin E_2_ are secreted and enhance the inflammatory response (Surh et al. 2001; López-Bojórquez et al. 2004).

Although the inflammatory response is an important part of the immune system, if acute inflammation does not succeed in eradicating pathogens, the resulting chronic inflammation can cause a loss of organ function and a variety of diseases, including cancer, diabetes, autoimmune diseases, arthritis, and cardiovascular disease (Yoo et al. 2018; Singh et al. 2019). Therefore, in certain conditions, suppression of excessive inflammatory responses is important to reduce the occurrence of serious disease.

Numerous studies have focussed on anti-inflammatory botanical extracts with the potential for development into new drugs, as these extracts are safer and have lower toxicity than synthesized drugs. *Barringtonia* (Lecythidaceae) species are considered good candidate sources for botanical anti-inflammatory drugs. For example, *Barringtonia racemosa* L. has been reported to have anti-arthritic and anti-edoema activity (Osman et al. 2016; Patil and Patil 2017) and *B. acutangula* (L.) Gaertn. is used in traditional medicine to treat arthralgia, chest pain, dysmenorrhoea, inflammation, haemorrhoids, diarrhoea, and psychological disorders (Zafar Imam et al. 2012). In addition, fruits of *Barringtonia* plants exert antioxidant and anti-inflammatory effects in oedema and granuloma (Muralidhar et al. 2013; Patil and Patil 2017). However, no studies have investigated the anti-inflammatory activity of *B. angusta* Kurz, a traditional medicine from Vietnam. In this study, we investigated the potential anti-inflammatory activity of *B. angusta* methanol extract (Ba-ME) both *in vitro* and *in vivo* and explored the molecular mechanisms underlying its anti-inflammatory activity.

## Materials and methods

### Materials and reagents

Methanol extracts (95%) of *B. angusta* (leaves and stems)*, B. coccinea* (leaves and twigs)*, B. pauciflora* (leave, flowers, and twigs), and *B. racemosa* (leaves and twigs) were supplied by the Foreign Plant Extract Bank of the International Biological Material Research Centre (Daejeon, Korea; http://ibmrc.re.kr). *B. angusta* (leaf and stems) was collected on October 2017 from Phu Quoc National Park, Phu Quoc district, Kien Giang province, Vietnam. Plant samples were collected and identified by Dr. Tran from The Bach at the Institute of Ecology and Biological Resources (Hanoi, Vietnam). Voucher specimens, recorded as KRIB 0031699 and VK 3767, have been deposited at the herbarium of the Korea Research Institute of Bioscience and Biotechnology (Daejeon, Korea).

RAW264.7 cells and HEK293T cells were acquired from ATCC (Rockville, MD, USA). Roswell Park Memorial Institute 1640 (RPMI 1640) and Dulbecco's Modified Eagle's medium (DMEM) cell culture media, trypsin, and phosphate-buffered saline (PBS) were purchased from HyClone (Logan, UT, USA). Foetal bovine serum (FBS) was purchased from GIBCO (Grand Island, NY, USA).

Polyethylenimine (PEI), *N*-(1-naphthyl) ethylene-diamine dihydrochloride, ranitidine, sulphanilamide, LPS (*Escherichia coli* 0111:B4), Pam3CSK4, N(G)-nitro-L-arginine methyl ester (L-NAME), prednisolone (Pred), and 3-(4,5-dimethylthiazol-2-yl)-2,5-diphenyltetrazolium bromide (MTT) were purchased from Sigma Chemical Company (St. Louis, MO, USA). NF-κB luciferase constructs were obtained from Prof. Hae Young Chung (Busan National University, Busan, Korea). Antibodies against the phospho-specific or total forms of p65, p50/105, IκBα, Src, human influenza haemagglutinin *(*HA), and β-actin were purchased from Cell Signalling Technology (Beverly, MA, USA). TRI reagent® was acquired from Molecular Research Centre Incorporated (Cincinnati, OH, USA). Enhanced chemiluminescence (ECL) reagent was supplied from Amersham (Bath, UK). PCR primers specific for *iNOS*, *COX-2*, *TNF-α*, *IL-1β*, *IL-6*, and *GAPDH* were obtained from Macrogen (Seoul, Korea).

### Cell culture

RAW264.7 cells were cultured in RPMI1640 medium, and HEK293T cells were cultured in DMEM. Both media were supplemented with heat-inactivated FBS, glutamine, and antibiotics (penicillin and streptomycin). Cells were incubated at 37 °C under 5% CO_2_.

### Extract treatment

For *in vitro* treatment, a stock solution (100 mg/mL) of Ba-ME was dissolved in 100% dimethyl sulfoxide (DMSO) and diluted with culture medium. For *in vivo* experiments, Ba-ME was suspended in 0.5% sodium carboxymethylcellulose and administered at 50 and 100 mg/kg, twice per day for two days, to the mouse model.

### Animals

Institute of Cancer Research (ICR) mice (male, approximately 5–6 weeks old) were purchased from ORIENTBIO (Seongnam, Korea; www.orient.co.kr) and housed in cages (4 mice per cage) under a 12-h light/dark cycle at approximately 21–24 °C with 40% humidity. Mice were supplied 100 mL of water and 20 g of pelleted diet every day *ad libitum*. All experiments were performed in accordance with guidelines established by the Institutional Animal Care and Use Committee at Sungkyunkwan University (Suwon, Korea; approval ID: SKKUIACUC2019-06-21-1).

### NO production assay

RAW264.7 cells (1 × 10^6^ cells/mL) were plated in 96-well culture plates and incubated for 18 h. Ba-ME (0-150 µg/mL) or control compounds (L-NAME and Pred) was administered to the cells for 30 min, and then, the cells were treated with an inflammatory stimulus [LPS (1 µg/mL) or Pam3CSK (10 µg/mL)] for 24 h (Li et al. 2019). NO production was evaluated by analysing the level of colour reaction using Griess reagent, with the absorbance detected at 540 nm using a Spectramax 250 microplate reader.

### Cell viability test

RAW264.7 cells (1 × 10^6^ cells/mL) were plated and cultured for 18 h. Cells were treated with Ba-ME (0-150 µg/mL) or control compounds (Pred and L-NAME) and incubated for 24 h. Cell viability was evaluated using a conventional MTT assay, as reported previously (Han et al. 2018). MTT solution (10 μL of 10 mg/mL in PBS, pH 7.4) was added to the cell culture medium, and cells were incubated for 4 h. The reaction was then stopped by adding 15% sodium dodecyl sulphate (SDS) to each well to dissolve formazan crystals. Absorbance was detected at 570 nm using a Spectramax 250 microplate reader.

### High-performance liquid chromatography (HPLC) of Ba-ME

The phytochemical characteristics of Ba-ME were confirmed using HPLC analysis with silibinin, kaempferol, apigenin, genistein, quercetin, and naringenin as the standard compounds. The analysis used a system equipped with an HPLC (Jasco) and UV-Vis detector. Elution solvents were solvent A (2% acetic acid in H_2_O and 0.1% formic acid in MeOH:H_2_O = 10:90) and solvent B (0.5% acetic acid in H_2_O:acetonitrile = 50:50 and 0.1% formic acid in MeOH:H_2_O = 90:10). The gradient step of the solvent was from solvent A to solvent B, and a CAPCELL PAK C_18_ MG, 4.6 mm I.D. *×* 250 mm column was used (Alafiatayo et al. 2019).

### Quantitative reverse transcriptase-polymerase chain reaction (RT-PCR)

To evaluate mRNA levels of inflammatory genes (*iNOS*, *COX-2*, *IL-1β*, *TNF-α*, and *IL-6*), RAW264.7 cells were pre-treated with Ba-ME (0-150 µg/mL) for 30 min and incubated with LPS (1 μg/mL) for 6 h. Total mRNA was precipitated using TRI reagent^®^ according to the manufacturer's instructions. Complementary DNA was synthesized from 1 μg of total RNA using MuLV reverse transcriptase according to the manufacturer's instructions. PCRs were performed as previously reported (Choi, Kim et al. 2019). Results were normalized as the ratio of optimal density relative to that of GAPDH. Primer sequences are provided in Table 1.

**Table 1.** Semiquantitative PCR primer sequences (5′ to 3′).

Gene	Direction	Sequences (5**′** to 3**′**)
iNOS	Forward	CCCTTCCGAAGTTTCTGGCAGCAG
	Reverse	GGCTGTCAGAGCCTCGTGGCTTTGG
IL-1β	Forward	CAGGATGAGGACATGAGCACC
	Reverse	CTCTGCAGACTCAAACTCCAC
COX-2	Forward	CACTACATCCTGACCCACTT
	Reverse	ATGCTCCTGCTTGAGTATGT
TNF-α	Forward	TTGACCTCAGCGCTGAGTTG
	Reverse	CCTGTAGCCCACGTCGTAGC
IL-6	Forward	GTACTCCAGAAGACCAGAGG
	Reverse	TGCTGGTGACAACCACGGCC
GAPDH	Forward	CACTCACGGCAAATTCAACGGCA
	Reverse	GACTCCACGACATACTCAGCAC

### Plasmid transfection and luciferase reporter gene activity assay

HEK293T cells (1 × 10^6^ cells/mL) were transfected with the NF-κB-Luc plasmid (1 μg/mL) and the β-galactosidase plasmid (0.25 μg/mL) using the polyethylenimine (PEI) method. At 24 h after transfection, HEK293T cells were treated with Ba-ME (0–150 μg/mL) and incubated for another 24 h. Cells were harvested and lysed by freezing and thawing, and luciferase assays were performed using the Luciferase Assay System (Promega) as reported previously (Hong et al. 2018). For overexpression of genes, HEK293 cells (1 × 10^6^ cells/mL) were transfected with a plasmid driving the expression of HA-Src for 24 h. At 24 h after transfection, cells were treated with Ba-ME (0–150 μg/mL) for 24 h. The expression of inflammatory response-related proteins were determined by Western blotting analysis.

### Western blotting analysis

Protein extracts (20 µg) from cell lysates were electrophoresed on SDS-polyacrylamide gels (Tris-base, 10% SDS, 30% acrylamide, 10% APS, TEMED, pH 8.8) using running buffer (Tris-base, 10% SDS, glycine). Separated proteins were transferred to a polyvinylidene difluoride membrane using transfer buffer (Tris-base, glycine, 10% SDS, methanol). Membranes were blocked with 3% bovine serum albumin (BSA) in TBST (Tris-base, NaCl, 0.1% Tween 20, pH 7.6) at room temperature for 1 h, followed by incubation with specific primary antibodies for 1 h. Membranes were then washed with 0.1% TBST three times for 10 min each, incubated with horseradish peroxide-linked secondary antibodies in 3% BSA for 1 h at room temperature and washed with 0.1% TBST three times for 10 min each. Target proteins were visualized using ECL reagent.

### Cellular thermal shift assay (CETSA)

HEK293T cells overexpressing Src were treated with Ba-ME (150 µg/mL) for 12 h and 1 *×* 10^5^ cells were sampled from each group. Cells were heated at one of five temperatures (47, 51, 55, 59, and 63 °C) for 3 min and cooled at room temperature for 3 min. Cells were lysed by 4 ∼ 5 freeze-thaw cycles using liquid nitrogen, followed by centrifugation at 14,000 rpm for 30 min. Next, 5*×* loading buffer was added to 60 µL of lysates, and samples were heated for 5 min at 95 °C. Protein expression levels were analysed by western blotting, with 20 µL of samples loaded into each well. Band intensities are related to protein stability and were quantified using ImageJ software (National Institute of Health, Bethesda, MD, USA).

### HCl/EtOH-induced gastritis mouse model

To establish the HCl/EtOH-induced gastritis mouse model, mice were fasted for 24 h. Mice were orally treated with Ba-ME (50 and 100 mg/kg) or ranitidine twice per day for two days. At 8 h after the final oral injection, acute gastritis was induced following a previously described protocol (Kim et al. 2015; Sun et al. 2017); all groups except the normal group were orally treated with 300 µL of 150 mM HCl/60% EtOH for 1 h. At 1 h after HCl/EtOH administration, the stomachs of all mice were excised along the greater curvature, and inflammatory lesions were measured using ImageJ software.

### Preparation of cellular and tissue lysates

Prepared RAW264.7 cells and HEK293T cells were washed with cold PBS and lysed with lysis buffer (20 mM Tris-HCl, pH 7.4, 2 mM EDTA, 2 mM EGTA, 50 mM glycerol phosphate, 2 μg/mL aprotinin, 2 μg/mL leupeptin, 1 μg/mL pepstatin, 50 μM PMSF, 1 mM benzamide, 10% glycerol, 0.1 mM sodium vanadate, 1.6 mM pervanadate). To ensure adequate lysis, cells were frozen in lysis buffer at −70 °C for 3 h. Total cell lysates were centrifuged at 12,000 rpm for 10 min at 4 °C and stored at −70 °C until use. To prepare stomach tissue lysates, stomach tissues were ground, lysed with lysis buffer and centrifuged at 12,000 rpm for 10 min at 4 °C. The supernatant was transferred to new tubes.

### Statistical analysis

The results in this study are presented as the mean ± standard deviation of at least three experiments. For statistical comparison, analysis of variance (ANOVA)/Scheffe’s *post hoc* test or the Kruskal–Wallis/Mann–Whitney test were applied to the data. *p*-Values < 0.01 or < 0.05 were considered statistically significant depending on the comparison group and statistical test.

## Results

### *In vitro* anti-inflammatory activity of Ba-ME and HPLC analyses

To evaluate the ability of Ba-ME to regulate inflammatory responses, we examined the production of NO in RAW264.7 cells stimulated by LPS (a TLR4 ligand) or Pam3CSK4 (a TLR2 ligand) and treated with Ba-ME compared with control compounds. NO production was strongly suppressed by Ba-ME in LPS- and Pam3CSK4-induced RAW264.7 cells, with IC_50_ values of 123.33 µg/mL and 46.89 µg/mL, respectively (Figure 1(A)). Control drugs [Pred (150 and 200 µM) and the iNOS inhibitor L-NAME (0.5, 1, and 1.5 mM)] dose-dependently reduced NO production under Pam3CSK- and LPS-treated conditions (Figure 1(A) right panel, 1 (F) left panel, and 1 (G) left panel) without displaying cytotoxicity (Figure 1(F) right panel and 1 (G) right panel). To examine whether suppression of NO affected cell viability, we performed cell viability assays and confirmed that Ba-ME treatment caused no cell cytotoxicity at concentrations up to 150 µg/mL (Figure 1(B)). To compare the effects of Ba-ME among extracts from the same genus (*B. coccinea, B. pauciflora,* and *B. racemosa*), we repeated the anti-inflammatory experiments with these extracts and found that Ba-ME suppressed NO production to a greater level than the other extracts and was the least cytotoxic at all concentrations evaluated (Figure 1(C)).

Figure 1.Anti-inflammatory effects of Ba-ME in activated RAW264.7 cells and phytochemical analysis of Ba-ME. (A, F left panel, and G left panel) RAW264.7 cells were activated with LPS (1 µg/mL) and Pam3CSK4 (10 µg/mL) and then administered Ba-ME (0–150 µg/mL) or the control drug [L-NAME or prednisolone (Pred)]. NO production was measured using the Griess assay. (B, F right panel, and G right panel) RAW264.7 cells were treated with Ba-ME (0–150 µg/mL) or control drug (Pred and L-NAME), and cell viability was measured by the MTT assay. (C) To compare the anti-inflammatory effects of extracts, NO production and cell viability were analysed using various concentrations of Ba-ME and extracts from other *Barringtonia* plants. (D) Phytochemical characteristics of Ba-ME were analysed by HPLC using standard compounds (silibinin, kaempferol, apigenin, genistein, quercetin, and naringenin), with the flavonoid contents calculated using the curves of the standard compounds. (E) The NO inhibitory activity of silibinin was evaluated using LPS-treated RAW264.7 cells in the presence and absence of silibinin (6.25 and 12.5 µg/mL) for 24 h. ##*p* < 0.01 compared with the normal group, and **p < 0.01 compared with the control group.

**Figure:**
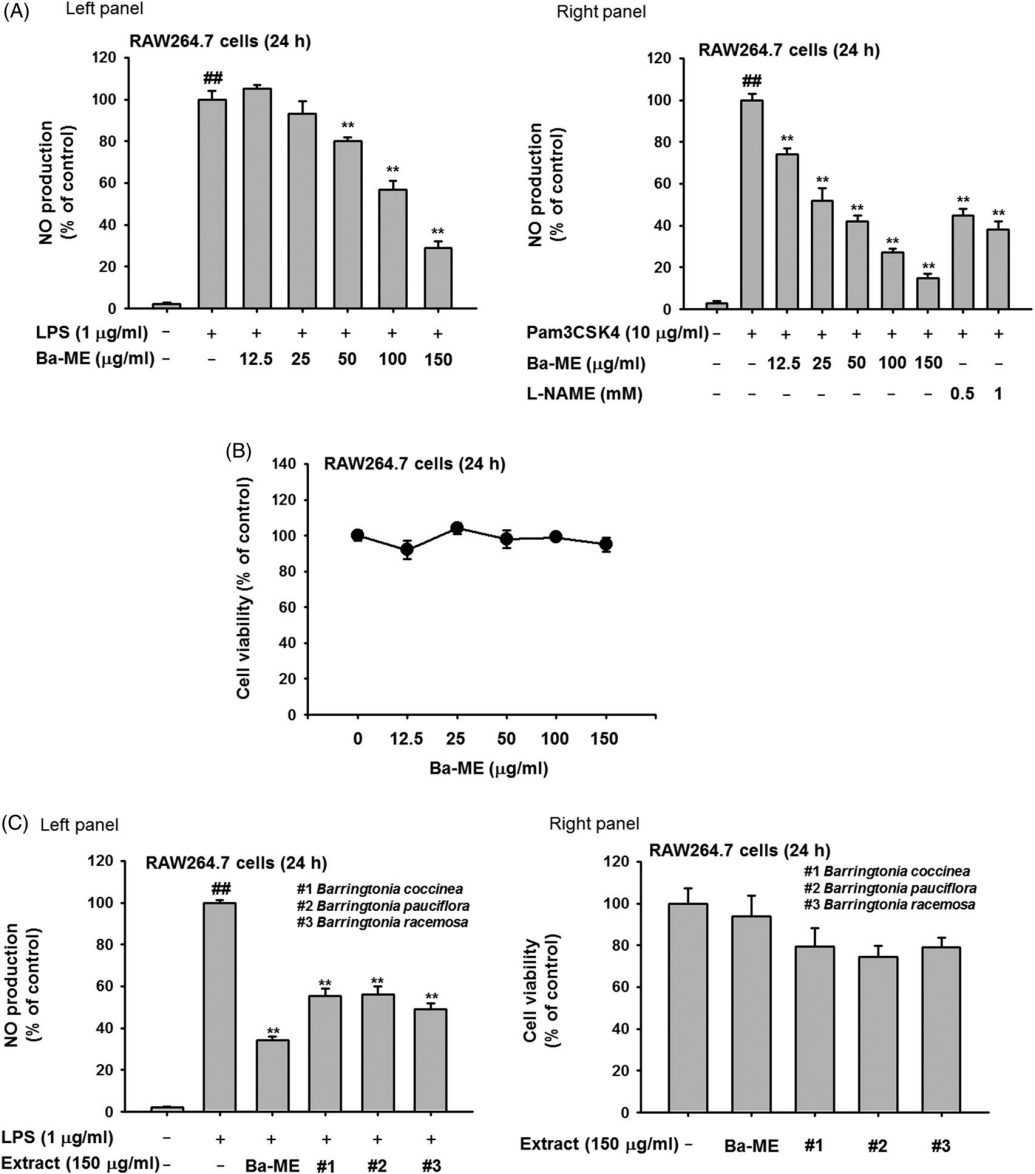

**Figure:**
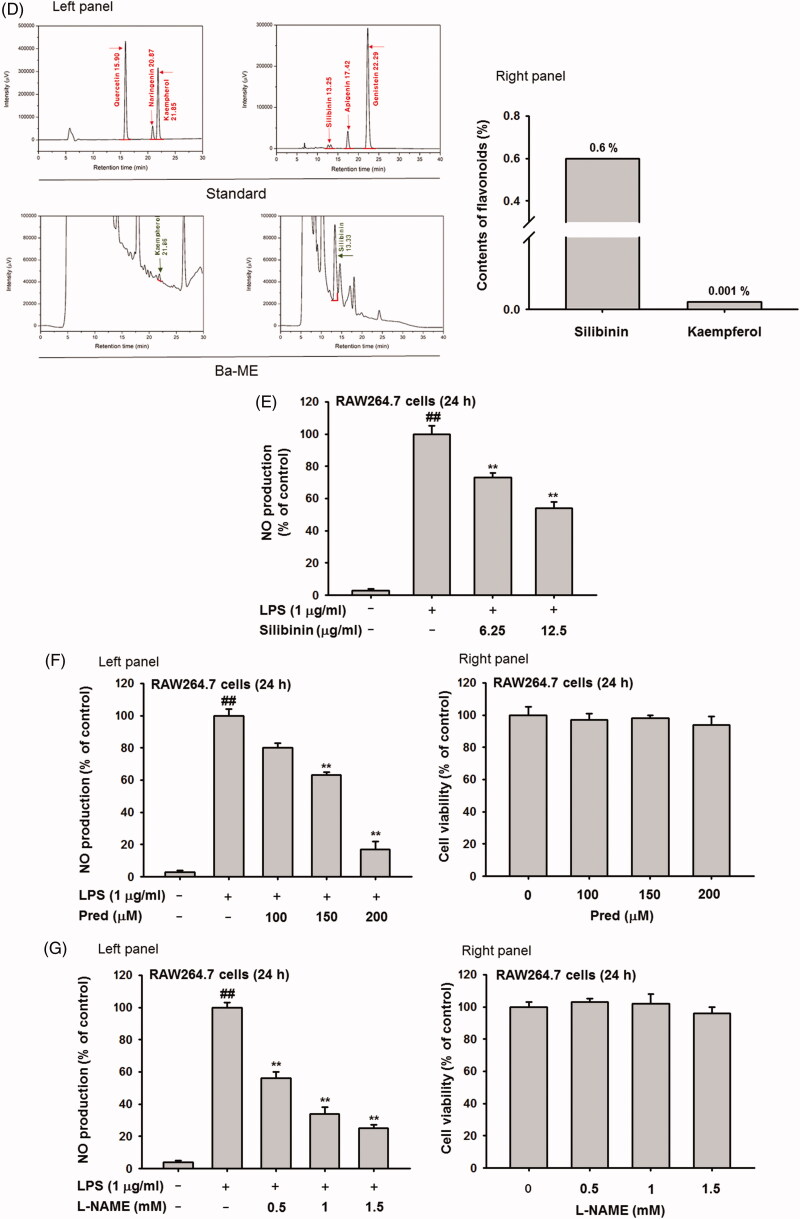

To identify the anti-inflammatory components in Ba-ME, we conducted HPLC with standard flavonoids known to have strong anti-inflammatory effects: silibinin, kaempferol, apigenin, genistein, quercetin, and naringenin (Comalada et al. 2005; Shi Hyoung et al. 2015; Zheng et al. 2017). The resulting HPLC profile revealed 3016 ppm of total flavonoids (silibinin and kaempferol), with 6.032 mg of flavonoids per 1 g of Ba-ME. The extract comprised 0.6% of silibinin (3009 ppm) and 0.001% of kaempferol (6.9 ppm), and we thus hypothesized the anti-inflammatory activity of Ba-ME may be mediated by those flavonoids (Figure 1(D)). To investigate the effects of silibinin on NO production, we examined NO levels in LPS-activated RAW264.7 cells exposed to silibinin. As shown in Figure 1(E), silibinin (6.25 and 12.5 µg/mL) reduced NO production up to 24.6% and 43.2%, respectively. However, silibinin (0.57% to 0.6%) was found to be included in Ba-ME with 0.80 to 1 µg/mL at 150 µg/mL by phytochemical analysis, implying that this compound is not the active component in Ba-ME.

Suppressive effect of Ba-ME on inflammatory gene expression and activation of transcription factors.

**Figure 2.**
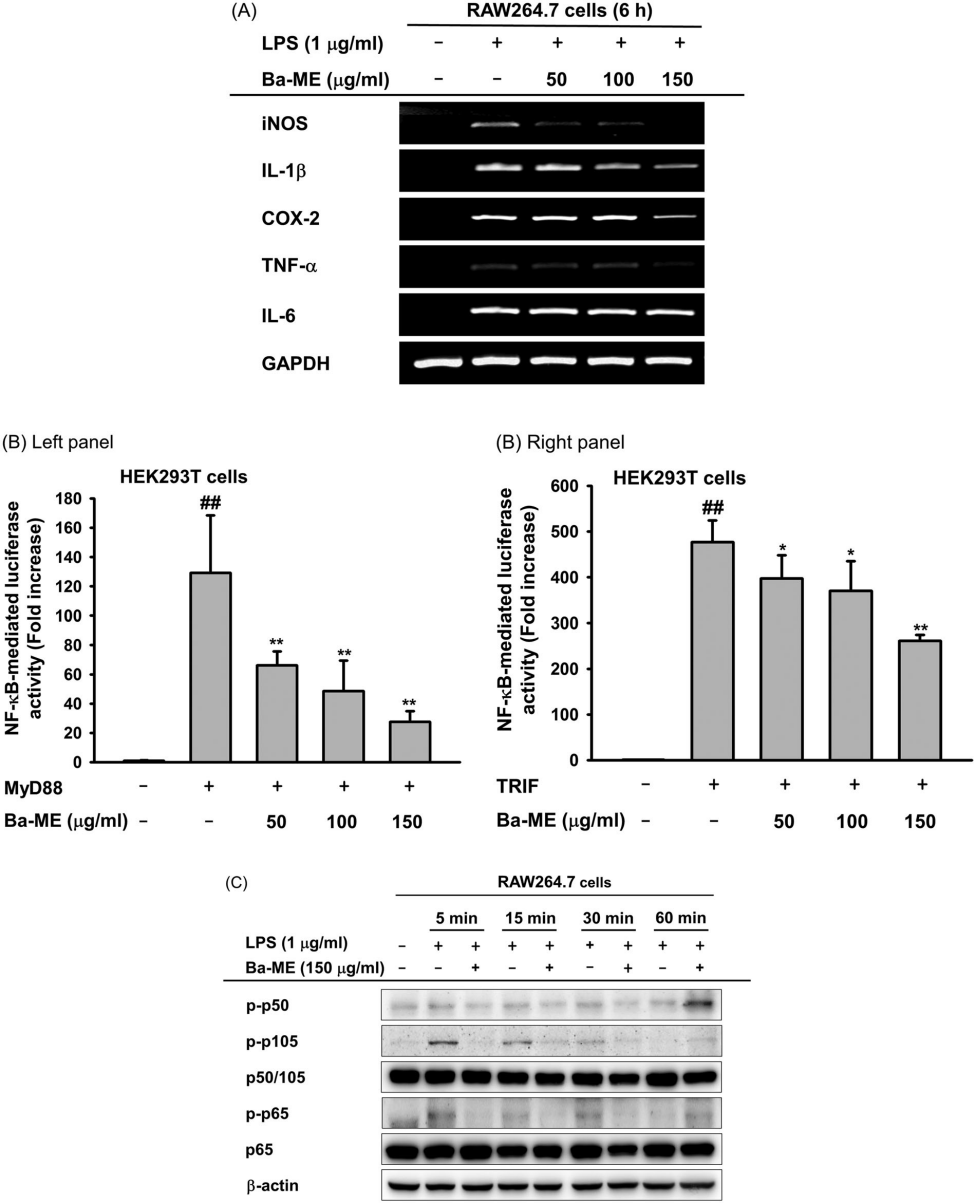
Expression of inflammatory genes and regulation of transcription factors after Ba-ME treatment. (A) mRNA expression levels of iNOS, COX-2, TNF-α, IL-1β, and IL-6 were determined by semiquantitative RT-PCR. (B) HEK293T cells were co-transfected with the NF-κB luciferase construct, MyD88 or TRIF plasmids, and a β-gal plasmid (as a transfection control). Cells were treated with Ba-ME (0–150 µg/mL) for 24 h. Luciferase activity was determined using a luminometer. (C) Expression levels of the phosphorylated forms of p50 and p65 in RAW264.7 cells were examined by western blotting. ##*p* < 0.01 compared with the normal group; *p < 0.05 and **p < 0.01 compared with the control group.

To further investigate the anti-inflammatory activity of Ba-ME, we examined inflammatory gene expression levels at the transcriptional level and the nuclear translocation of transcription factors. Transcript levels of inflammatory genes were upregulated in LPS-stimulated RAW264.7 cells, while Ba-ME significantly reduced the expression of *iNOS*, *IL-1β*, *COX-2*, *TNF-α*, and *IL-6* (Figure 2(A)).

To examine the transcriptional activation level of NF-κB, a key transcription factor in inflammation, we performed luciferase reporter gene assays using MyD88- or TRIF-induced HEK293T cells. Transcription of NF-κB was highly upregulated by MyD88 and TRIF and suppressed by 50, 100, and 150 µg/mL of Ba-ME (Figure 2(B)). Because transcriptional activation of NF-κB was reduced by Ba-ME, we next investigated translocation of the p65 and p50/105 NF-κB subunits by assessing levels of phosphorylated p65 and p50/105 by Western blot analysis of whole cell lysates of LPS-stimulated RAW264.7 cells. Cells were stimulated with LPS for 5–60 min, which resulted in upregulation of phosphorylation of p65 and p50/105; treatment with Ba-ME suppressed the increase in phosphorylation of these subunits at an early time point (Figure 2(C)). These results indicate that Ba-ME suppressed gene transcription of NF-κB and translocation of NF-κB protein to the nucleus. Furthermore, because suppression occurred early during NF-κB signalling, we hypothesised that Ba-ME targets upstream molecules of the NF-κB pathway.

Ba-ME binds to the Src-SH2 domain and regulates the kinase activity of Src.

We next examined the effect of Ba-ME on the activation of NF-κB signalling by evaluating the phosphorylation levels of Src and IκBα, which are involved in NF-κB activation (Chen et al. 2003; Kang et al. 2006). While phosphorylated Src and IκBα in RAW264.7 cells increased with LPS stimulation, Ba-ME treatment dramatically reduced levels of phosphorylated Src and IκBα (Figure 3(A)).

Figure 3.Ba-ME targets Src in the NF-κB signalling pathway. (A) RAW264.7 cells were treated with Ba-ME (150 µg/mL) and LPS (1 µg/mL), and levels of total and phosphorylated IκBα and Src were detected by western blotting. (B) HEK293T cells overexpressing HA-Src were treated with Ba-ME (0–150 µg/mL) for 24 h. Total and phospho-forms of Src and β-actin were examined by western blotting. (C) Cellular thermal shift assay (CETSA) was performed using HEK293T cells overexpressing Src that were treated with Ba-ME (150 µg/mL) or DMSO (vehicle solution). Src protein stabilization was examined by western blotting, and band intensities were measured using ImageJ software. (D) Image of the Src domain. (E) Plasmids expressing wild-type Src and Src-SH2 and -SH3 domain deletion mutants were transfected into cells that then were treated with Ba-ME for 24 h. Phosphorylated Src and the HA-tagged protein were detected by western blotting, and band intensities are presented. (F) (shown below) CETSA performed using HEK293T cells expressing the Src-SH2 domain-deleted mutant treated with Ba-ME (150 µg/mL) or DMSO (vehicle solution). Src protein stability was examined by western blotting, and band intensities were measured using ImageJ software.

**Figure:**
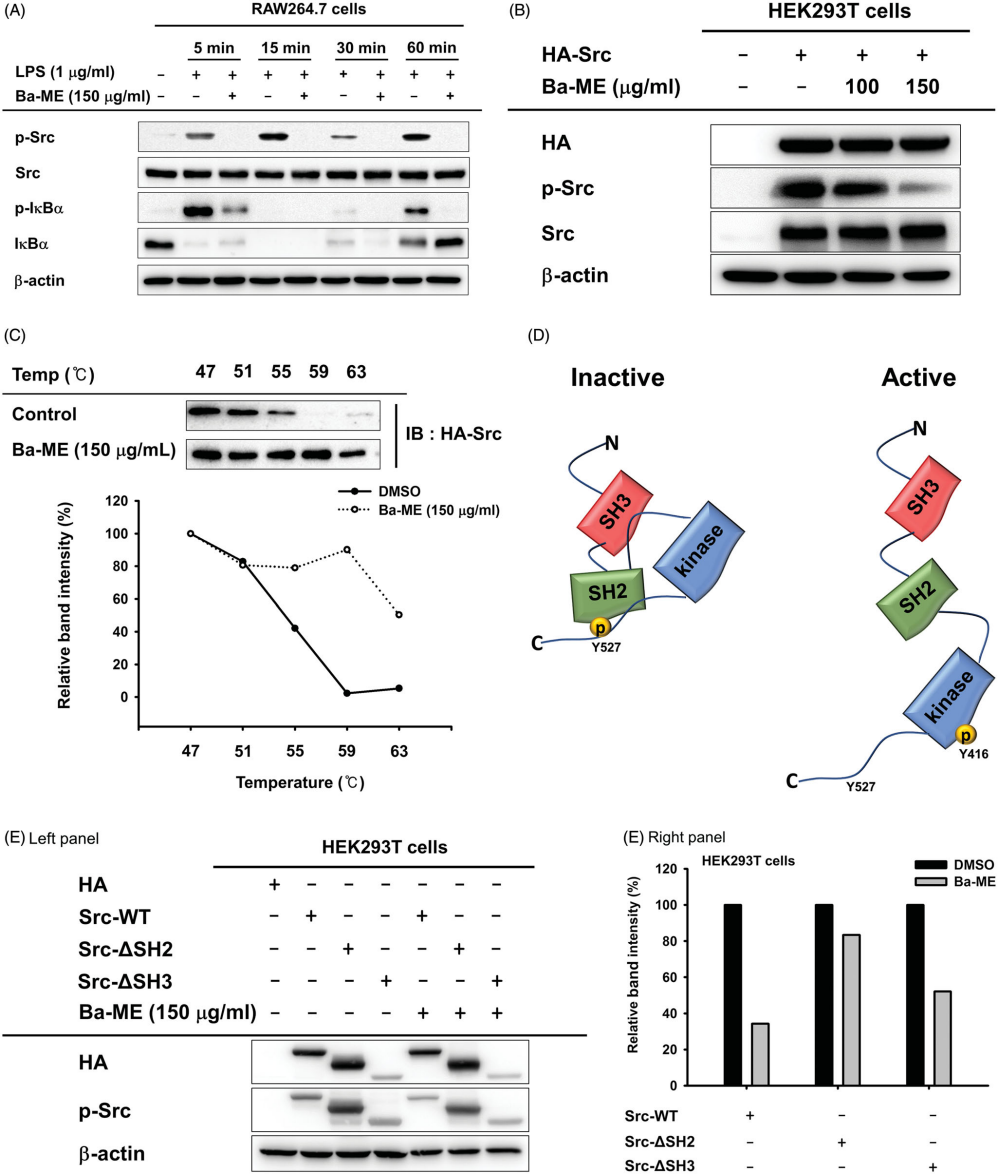

**Figure:**
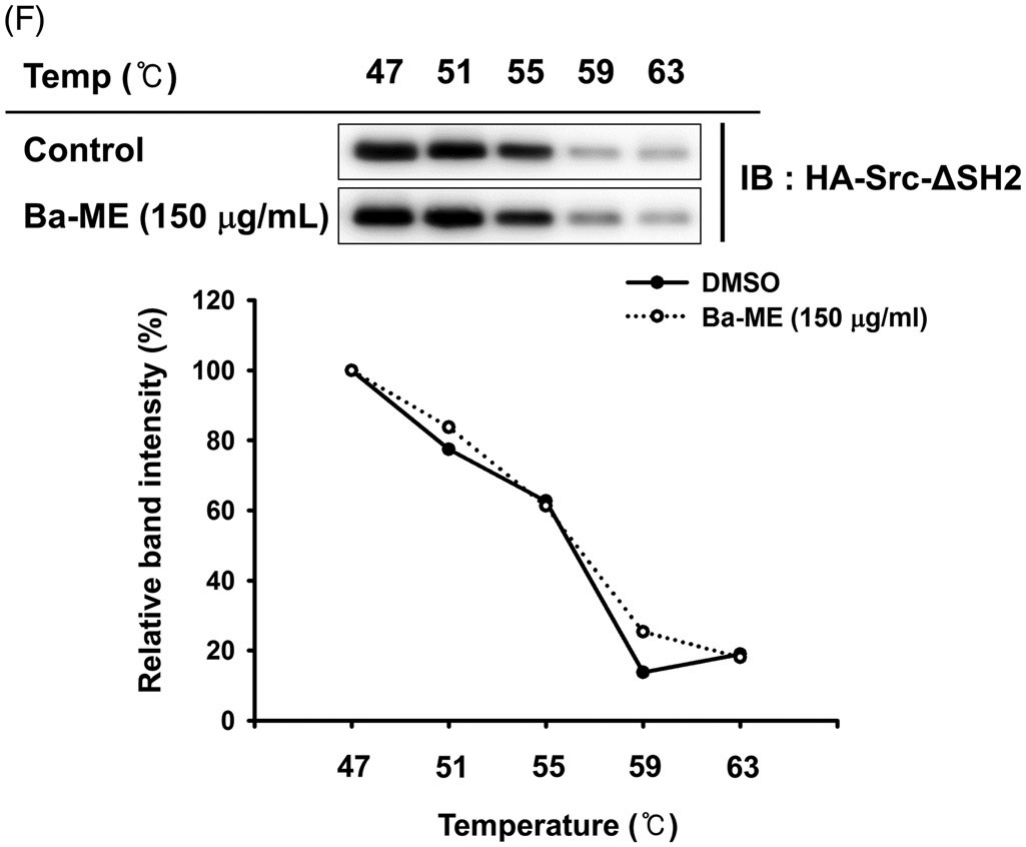

Based on the immunoblotting results, we hypothesized that Src is the target of Ba-ME. Therefore, we overexpressed Src in HEK293T cells and found that the level of phosphorylated Src was decreased by Ba-ME treatment (Figure 3(B)). We next performed CETSA to examine the interaction between Ba-ME and Src; this assay is based on the principle that thermal stabilization of a protein is maintained by ligand binding (Jafari et al. 2014). HEK293T cells overexpressing Src were treated with Ba-ME or vehicle and exposed to temperatures ranging from 47 °C to 63 °C. Src was degraded at temperatures of 59 °C–63 °C in the vehicle (DMSO)-treated group; however, in the Ba-ME-treated group, Src was stable at these elevated temperatures, indicating that Ba-ME binds to the Src protein (Figure 3(C)).

To investigate whether Ba-ME regulates Src kinase activity, we overexpressed Src mutants deleted for the SH2 or SH3 domains (Src-dSH2 and Src-dSH3, respectively) affecting structural feature of Src (Figure 3(D)) and found that the level of phosphorylated Src was reduced in HA-Src-WT- and HA-Src-dSH3-overexpressing cells, whereas the level of phosphorylated Src did not change in HA-Src-dSH2-overexpressing cells (Figure 3(E)). Moreover, in CETSA, Src lacking the SH2 domain was not stable, suggesting that Ba-ME binds to the Src-SH2 domain (Figure 3(F)). These results indicate that Ba-ME targets the Src protein and regulates Src activity by binding to its SH2 domain.

*In vivo* anti-inflammatory effects of Ba-ME in an HCl/EtOH-induced acute gastritis mouse model

**Figure 4.**
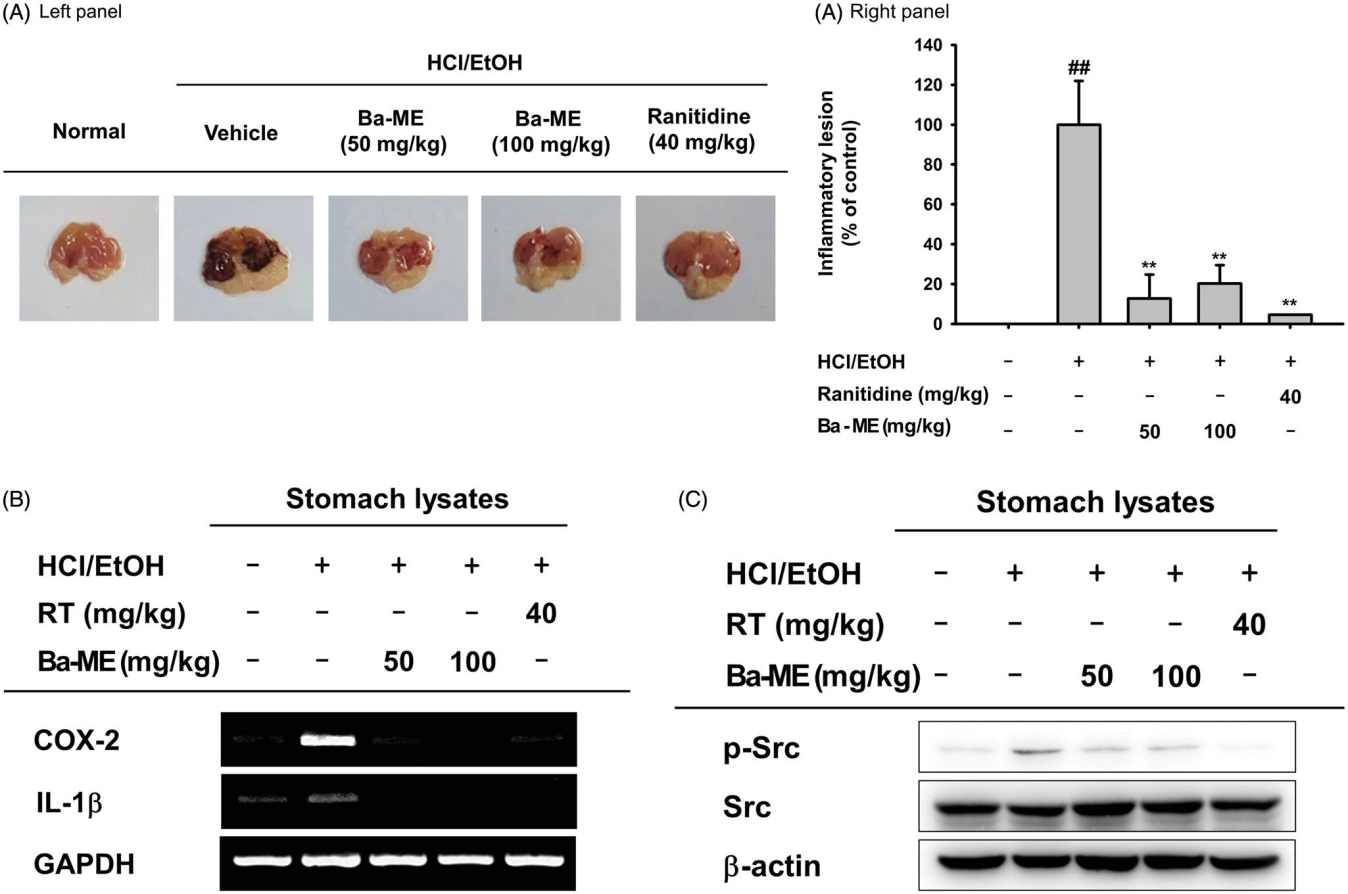
Anti-inflammatory effect of Ba-ME on HCl/EtOH-induced gastritis in mice. (A) Mice were orally injected with Ba-ME (50 and 100 mg/kg) or ranitidine (40 mg/kg) four times before oral administration of HCl/EtOH. At 1 h after administration of HCl/EtOH, the stomachs of the mice were excised, and gastric lesions in the stomachs were measured with ImageJ. The gastritis index of the control group (inducer alone) is represented as 100%. (B) mRNA expression levels of COX-2 and IL-1β in the stomach tissues of mice treated with HCl/EtOH were determined by semiquantitative RT-PCR. (C) Levels of the total and phosphorylated forms of Src in stomach tissues of mice treated with HCl/EtOH were examined by western blotting. ##*p* < 0.01 compared with the normal group and **p < 0.01 compared with the control group.

To examine the anti-inflammatory effect of Ba-ME *in vivo*, we used an HCl/EtOH-induced acute gastritis mouse model. Ba-ME (50 and 100 mg/kg) was orally administered before administration of HCl/EtOH. The mice were then sacrificed and the area of ulceration in the stomach was evaluated. The group that received Ba-ME had fewer inflammatory lesions than the group that did not receive Ba-ME. Furthermore, treatment with Ba-ME reduced gastritis to the same extent as treatment with ranitidine, which is a standard anti-inflammatory agent (Figure 4(A)).

We used RT-PCR to analyse gene expression and immunoblotting to examine protein expression in the lysates of excised stomachs. Ba-Me treatment decreased the transcript levels of *COX-2* and *IL-1β* and protein levels of phosphorylated Src (Figure 4(B and C)). Together these results showed that Ba-ME ameliorated gastritis and suppressed Src activity and the expression of inflammatory genes *in vivo*.

## Discussion

Diverse botanical extracts are being evaluated in drug development efforts for various diseases because these extracts have potent biological activity with fewer side effects and lower toxicity compared with synthesised agents. To identify potential drug candidates with anti-inflammatory effects, we checked the inhibitory activity of *Barringtonia* spp. extracts using the NO production assay and found that 150 µg/mL of Ba-ME significantly suppressed NO production. Therefore, we studied the anti-inflammatory effects of Ba-ME. While previous research has reported anti-inflammatory effects for extracts from other plants in the same genus, here we demonstrated the superiority of Ba-ME in suppressing inflammation compared with the other extracts in comparative experiments (Figure 1(C)) (Behbahani et al. 2007).

In this study, we evaluated the anti-inflammatory effects of Ba-ME in various ways. Several studies have indicated that the anti-inflammatory effects of botanical extracts and plants are due to specific anti-inflammatory flavonoids, such as luteolin, kaempferol, quercetin, and silibinin (Comalada et al. 2005; Shi Hyoung et al. 2015; Zheng et al. 2017; Aziz et al. 2018). Our HPLC analysis revealed that Ba-ME contains silibinin and kaempferol, with particularly higher silibinin levels. However, our experiments showed that silibinin is not a key contributor to the anti-inflammatory activity of Ba-ME, since this compound is present in the extract at a level too low to show NO inhibitory activity (Figure 1(D,E)), as shown in previous reports (Giorgi et al. 2012; Youn et al. 2013). For *in vitro* analyses, we used activated RAW264.7 cells to investigate how Ba-ME attenuates inflammatory responses. NO production plays a key role in inflammation (Tripathi et al. 2007), and we confirmed that Ba-ME inhibits NO production in macrophages induced by specific ligands of TLR4 and TLR2, namely LPS and Pam3CSK4, respectively (Guzik et al. 2003; Nackiewicz et al. 2014) (Figure 1(A)). Similar results were found with the positive control drugs (Pred and L-NAME) (Figure 1(F,G)). Because Ba-ME suppressed NO production, we evaluated the mRNA expression of iNOS, which catalyses NO production. We also examined the mRNA expression of other inflammatory genes (COX-2, TNF-α, and IL-1β) in LPS-induced macrophages and found that Ba-ME regulated their expression at the transcriptional level. To investigate activation of inflammatory signalling pathways such as NF-κB, we performed a luciferase reporter gene assay and found that Ba-ME suppressed NF-κB-luciferase activity with induction of both MyD88 and TRIF (adaptor molecules of the TLR signalling pathway) (Figure 2(B)) (Yamamoto et al. 2003). We thus hypothesized that Ba-ME may suppress the nuclear translocation of NF-κB subunits (p50 and p65) and found that levels of phosphorylated p50 and p65 were decreased by Ba-ME treatment (Figure 2(C)). Therefore, Ba-ME suppresses the translocation of active NF-κB into cell nuclei and affects the transcriptional activity of NF-κB (Christian et al. 2016).

Based on our findings showing that NF-κB translocation and activity were suppressed by Ba-ME, we investigated molecules upstream of NF-κB and found that level of phosphorylated IκBα, which is degraded to release p50 and p65, was decreased by Ba-ME treatment of cells exposed to LPS for 5, 15, 30, or 60 min. Our findings showed that inhibition occurred at an early time point, and therefore, we predicted that Ba-ME targeted upstream molecules in the NF-κB signalling pathway. Activation of tyrosine kinases is important during macrophage activation and is related to inflammatory cytokine production, indicating that Src can be targeted to regulate inflammatory responses (Smolinska et al. 2008). Several studies have reported the anti-inflammatory effect of extracts that target the kinase activity of Src protein (Thai et al. 2015; Kim, Choi et al. 2018; Choi, Cho, et al. 2019), which is primary kinase in early NF-κB signalling (Page et al. 2009). Src activity was strongly suppressed by Ba-ME after 5, 15, 30, and 60 min of LPS treatment. As Ba-ME inhibits the inflammatory response at an early stage, we predicted that Src was targeted by Ba-ME (Figure 3(A)) (Se Eun et al. 2012). To confirm that Src was a direct target of Ba-ME, HEK293T cells overexpressing Src were treated with 150 µg/mL Ba-ME, and the results showed that Src activity was suppressed (Figure 3(B)). CETSA results indicated that Ba-ME interacts directly with the Src protein, as reflected by maintenance of Src thermal stability in response to increasing temperature (Figure 3(C)) (Jafari et al. 2014). Together, these results indicate that Ba-ME is an effective anti-inflammatory drug that targets Src kinase activity.

Dephosphorylation of Src-Y527 weakens the binding between the SH3 domain and the SH2-kinase linker and separates the SH2 domain and C-tail, producing the open form of Src. This open formation facilitates phosphorylation of Src-Y416, a key residue involved in kinase activity (Figure 3(D)) (Arold et al. 2001; Irtegun et al. 2013). We further investigated which domain of Src actively interacts with Ba-ME. Ba-ME suppressed Src kinase activity when the SH3 domain of Src was deleted, indicating that Src regulation by Ba-ME is based on the Src-SH2 domain (Figure 3(E)). Moreover, we conducted a CETSA experiment using the Src-SH2 domain deletion mutant and found no difference between Ba-ME-treated and untreated groups, suggesting that Ba-ME stabilizes Src in response to elevated temperatures by binding to the Src-SH2 domain. Therefore, we suggest that Ba-ME suppresses the NF-κB-mediated inflammatory response by binding to the SH2 domain of Src to downregulate its kinase activity.

We also examined the *in vivo* anti-inflammatory effect of Ba-ME in an HCl/EtOH-induced gastritis ICR mouse model. Ulcerative lesions in the stomachs of the Ba-ME-treated mice were dramatically ameliorated compared with those in non-treated mice (Figure 4(A)). Moreover, using stomach lysates from those mice, we confirmed that the mRNA expression of inflammatory genes, including COX-2 and IL-1β, was suppressed by Ba-ME treatment (Figure 4(B)). Because the anti-inflammatory effect of Ba-ME was mediated by Src *in vitro*, we also examined whether level of phosphorylated Src was downregulated in stomach tissues (Figure 4(C)). Our results are consistent with those of several other studies that have reported that anti-inflammatory extracts prepared from *Cerbera manghas* L. (Apocynaceae) and *Gouania leptostachya* DC. (Rhamnaceae) target Src, especially in the context of stomach inflammation (Jeong et al. 2014; To Thi Mai et al. 2015). Taken together, our results indicate that Ba-ME has *in vivo* anti-inflammatory activity mediated by regulation of Src kinase activity.

## Conclusions

**Figure 5.**
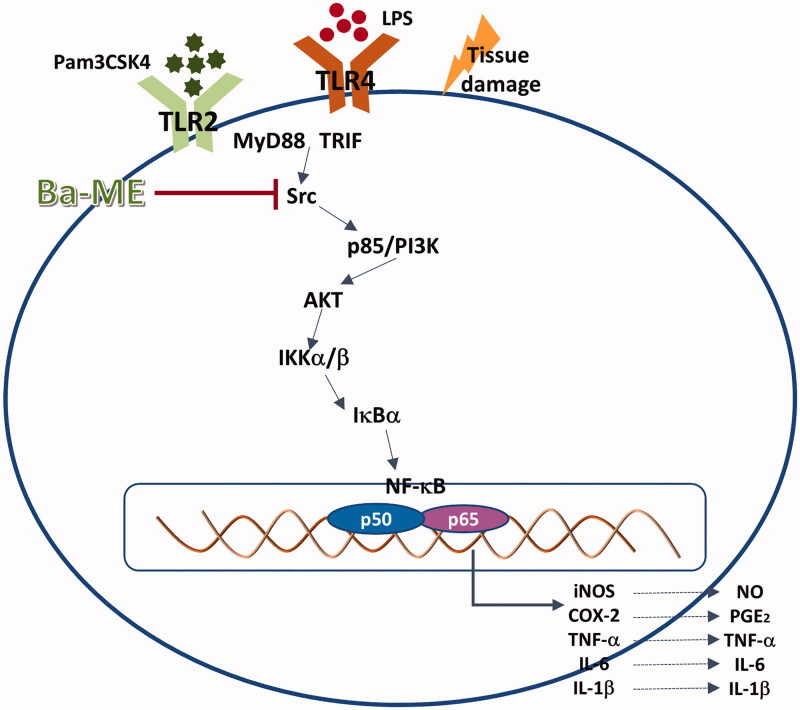
Molecular mechanisms underlying the anti-inflammatory action of Ba-ME. Ba-ME suppresses the phosphorylation of NF-κB pathway components and the translocation of p65 and p50 into the cell nucleus, thereby inhibiting inflammatory responses. LPS, lipopolysaccharide; TLR, toll-like receptor; MyD88, myeloid differentiation factor 88; TRIF, toll-receptor-associated activator of interferon; Ba-ME, *Barringtonia angusta* methanol extract; PI3K, phosphoinositide 3 kinase; IκBα, inhibitor of κBα..

We demonstrated that Ba-ME has *in vitro* and *in vivo* anti-inflammatory activity mediated by targeting of Src in the NF-κB signalling pathway (Figure 5). Furthermore, we found that Ba-ME interacts specifically with the SH2 domain of Src to inhibit Src kinase activity. These results suggest that Ba-ME is a promising anti-inflammatory drug candidate that targets Src in the NF-κB signalling pathway.
